# Supplementary material for: Realizing four-electron conversion chemistry for all-solid-state Li||I2 batteries at room temperature
Source: Nat Commun. 2025 Feb 18;16:1723. doi: 10.1038/s41467-025-56932-5 (PMC11836363; doi:10.1038/s41467-025-56932-5)
Supplement: Supplementary file 1 — Supplementary information [file 41467_2025_56932_MOESM1_ESM.pdf]

Supplementary Information

Realizing a four-electron solid-phase conversion chemistry for all-solid-state Li||I_2 batteries at room temperature

Zhu Cheng[1,2#], Hang Liu[1#], Menghang Zhang[1], Hui Pan[1], Chuanchao Sheng[1], Wei Li[1], Marnix Wagemaker[2], Ping He[1*] and Haoshen Zhou[1*]

[1]Center of Energy Storage Materials & Technology, College of Engineering and Applied Sciences, Jiangsu Key Laboratory of Artificial Functional Materials, National Laboratory of Solid State Microstructures and Collaborative Innovation Center of Advanced Microstructures, Nanjing University, Nanjing 210093 (P. R. China)

[2]Section Storage of Electrochemical Energy, Radiation Science and Technology, Faculty of Applied Sciences, Delft University of Technology, 2629 JB, Delft, The Netherlands

* E-mail: pinghe@nju.edu.cn; hszhou@nju.edu.cn

#These authors contributed equally.

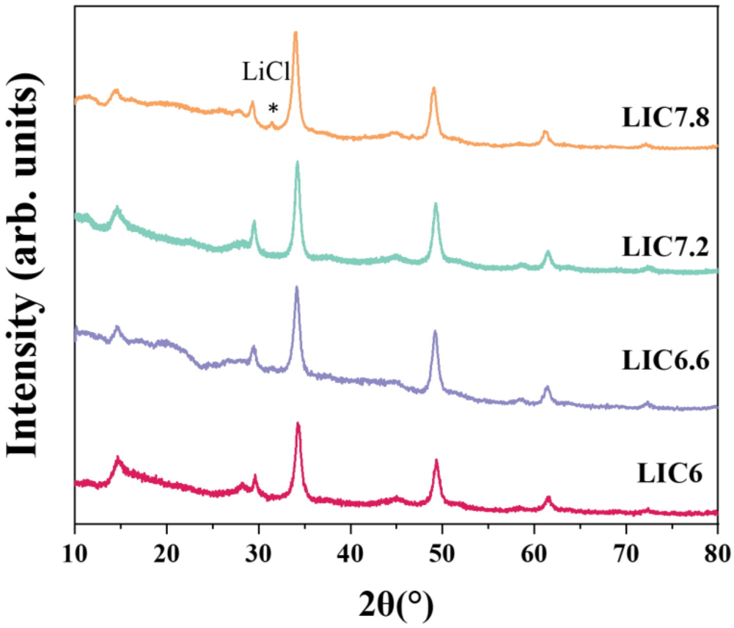

Supplementary Figure 1 | Phase analysis of ball-milled Li-In-Cl compounds with varying stoichiometry. XRD patterns of the ball milled LIC6, LIC6.6, LIC7.2 and LIC7.8. All the stoichiometries show a similar structure expect minor LiCl impurity is observed for LIC7.8. This indicates that the LIC7.8 is out of the Li-In-Cl solid solution range.

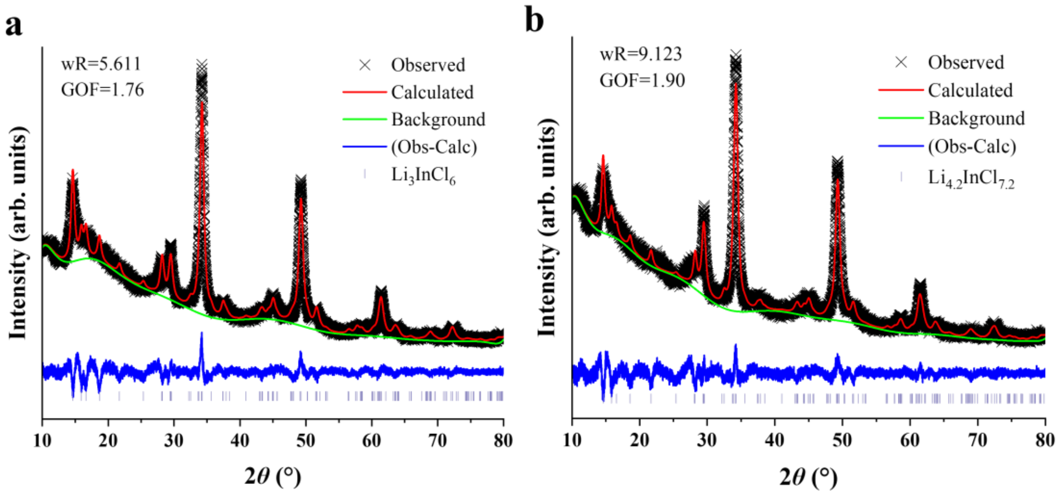

Supplementary Figure 2 | Rietveld refinement results of the ball milled (a) LIC6 and (b) LIC7.2.

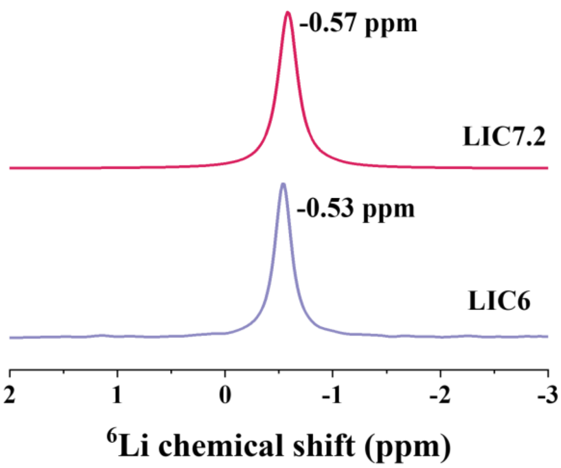

Supplementary Figure 3 | Nuclear magnetic resonance characterization of lithium environments. ^{6}Li NMR spectra of LIC6 and LIC7.2.

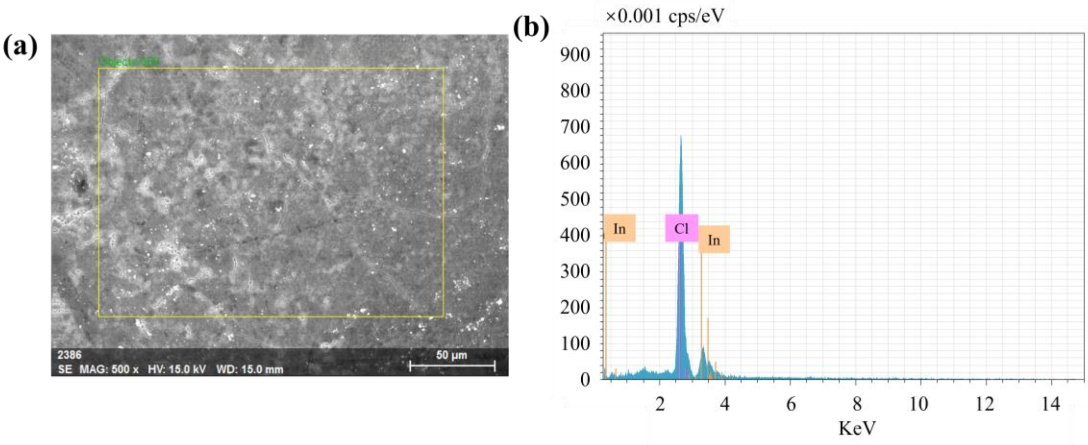

Supplementary Figure 4 | Elemental composition analysis of LIC7.2. (a) SEM image and (b) the element distribution information in the selected area of the LIC7.2 pellet.

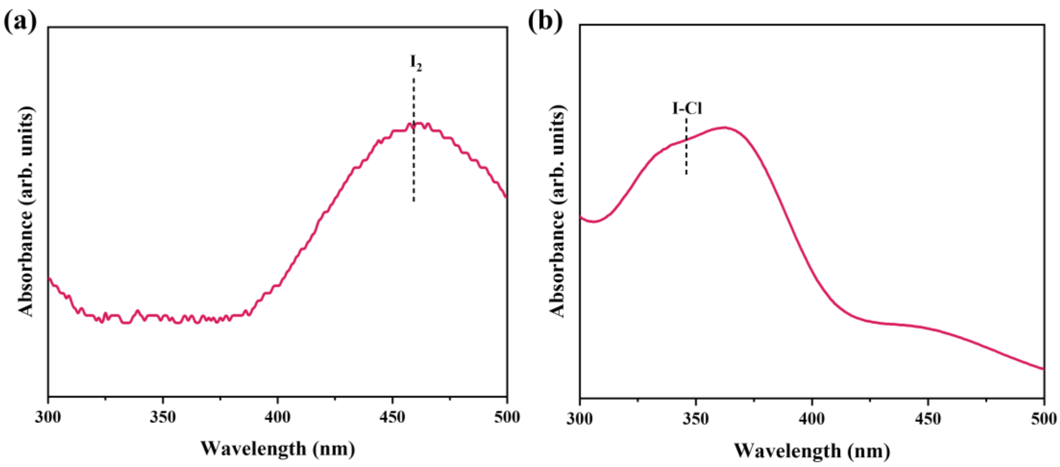

Supplementary Figure 5 | Solid-state UV vis spectrum testing of positive electrode. Solid-state UV vis spectrum of the I_2/LIC7.2 composite (a) at pristine and (b) after charging.

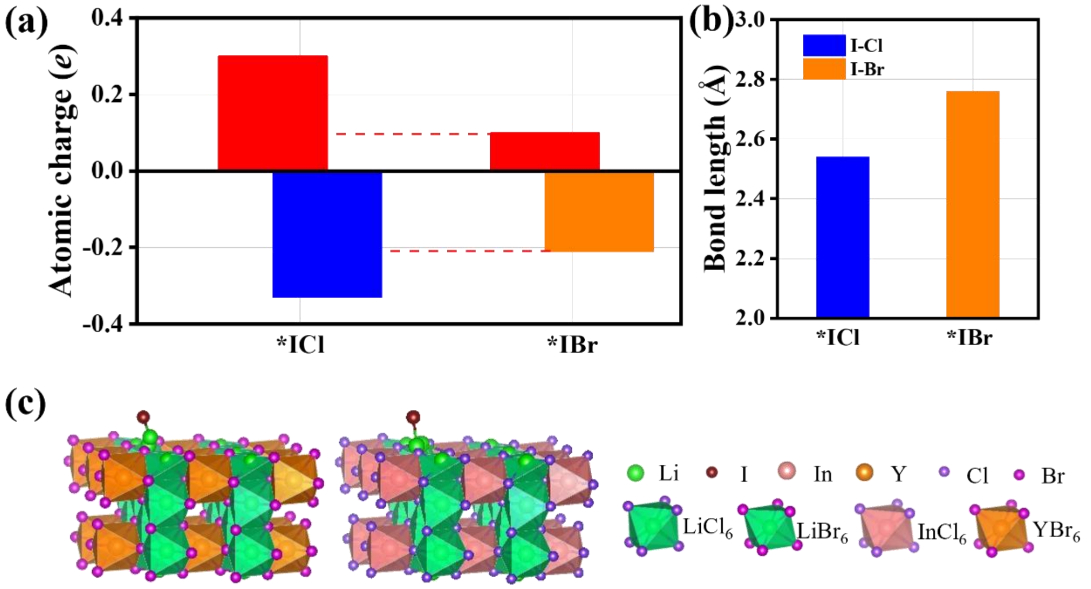

Supplementary Figure 6 | Computational analysis of halogen bonding characteristics. (a) Bader charge and (b) Bond length calculation results of *ICl in I_2/LIC7.2 and *IBr in I_2/LYB. (c) Inset shows the molecular structures used of LYB and LIC7.2 substrates used for DFT calculations.

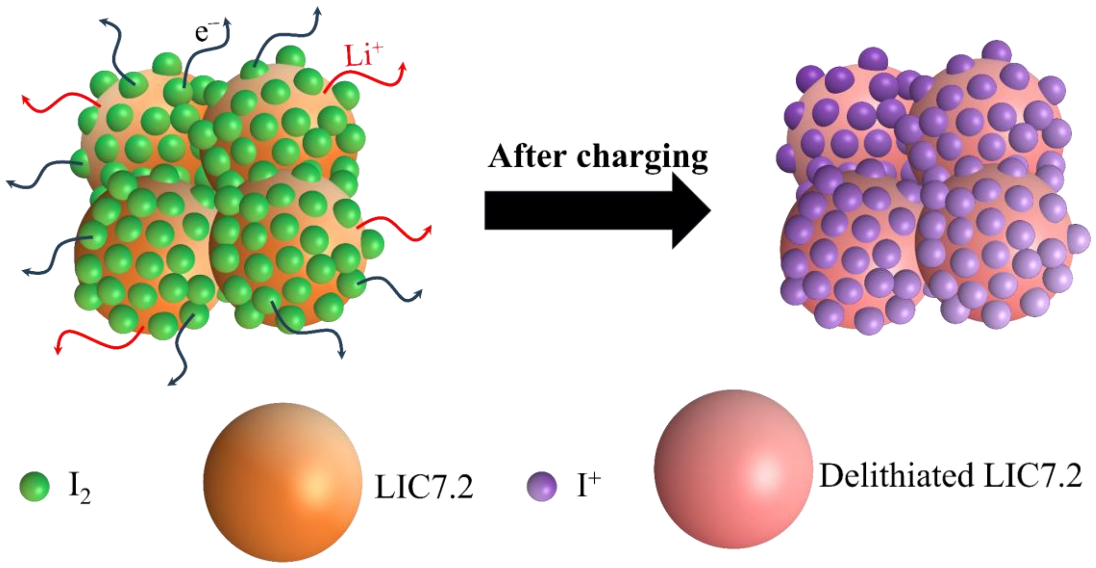

Supplementary Figure 7 | Proposed charging mechanism model for I2/LIC7.2 electrode system. Schematic of the charging reaction mechanism of I_2/LIC7.2 electrode. During charging, I_2 loses electrons and the LIC7.2 loses Li ion, then I^+ combines with LIC7.2 to form interhalogen bonds at the interface.

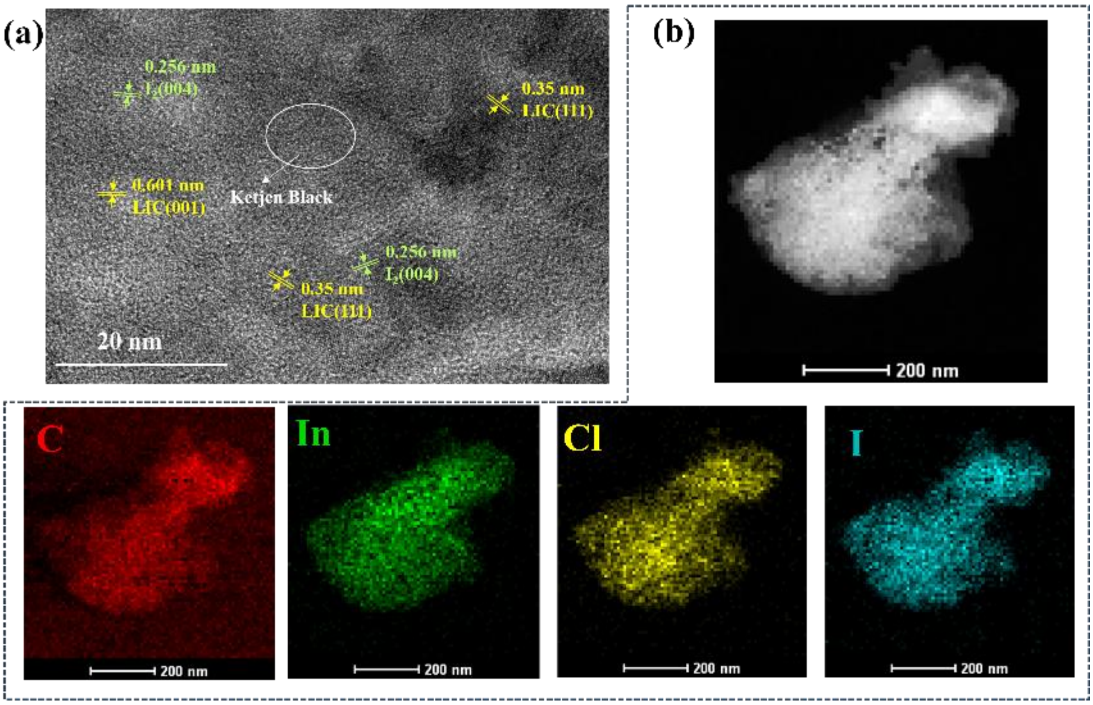

Supplementary Figure 8 | Nanoscale morphology and elemental distribution of composite material. (a) Transmission electron microscopy image of the ball milled I_2/LIC7.2 composite and (b) energy-dispersive X-ray mapping for different elements (C, In, Cl and I). It can be seen that the I_2 domain and LIC7.2 domain show a size of several nanometers.

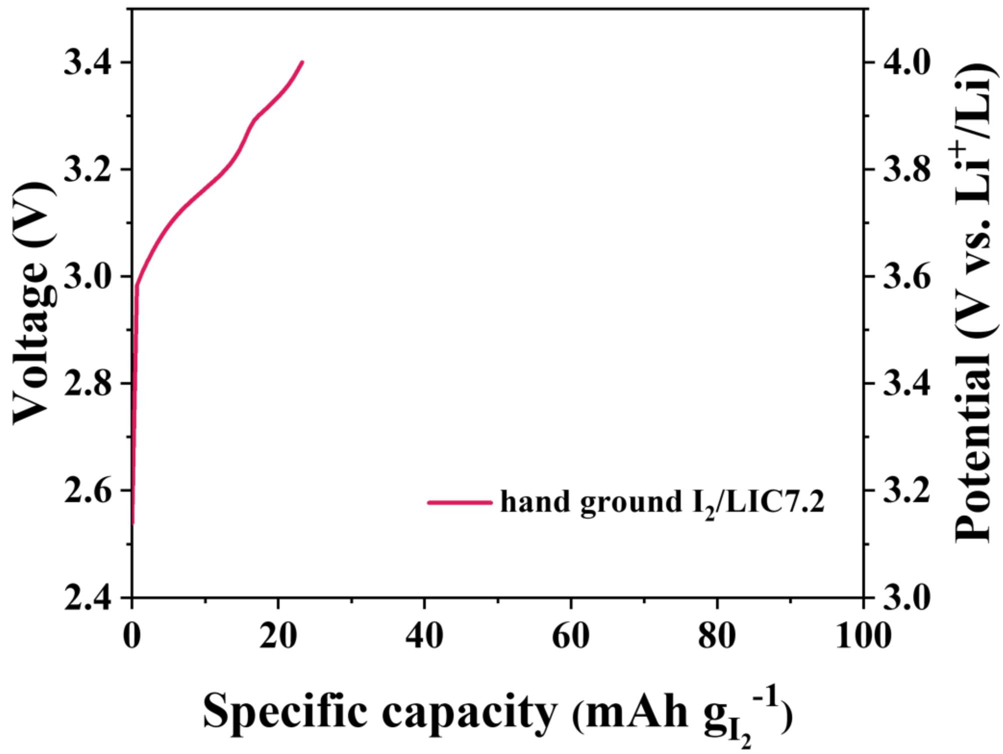

Supplementary Figure 9 | Electrochemical performance of hand ground electrode. Direct charging profile of the hand ground I_2/LIC7.2 electrode. The I_2 mass loading is 0.5 mg cm^{-2} and the test was performed at 44 mA g^{-1} and at 25°C.

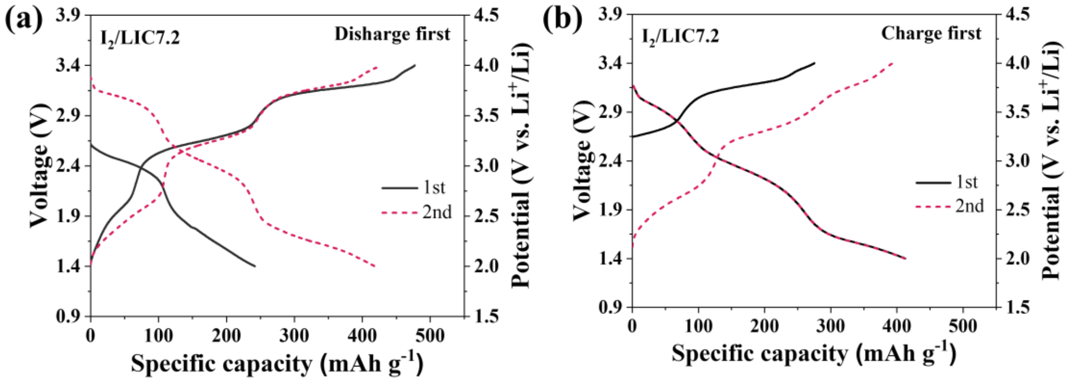

Supplementary Figure 10 | Initial cycling behavior under different discharge/charge sequences. Voltage profiles of first two cycles of the I_2/LIC7.2 battery (a) being discharged first and (b) being charged first at 44 mA g^{-1} and room temperature (25℃).

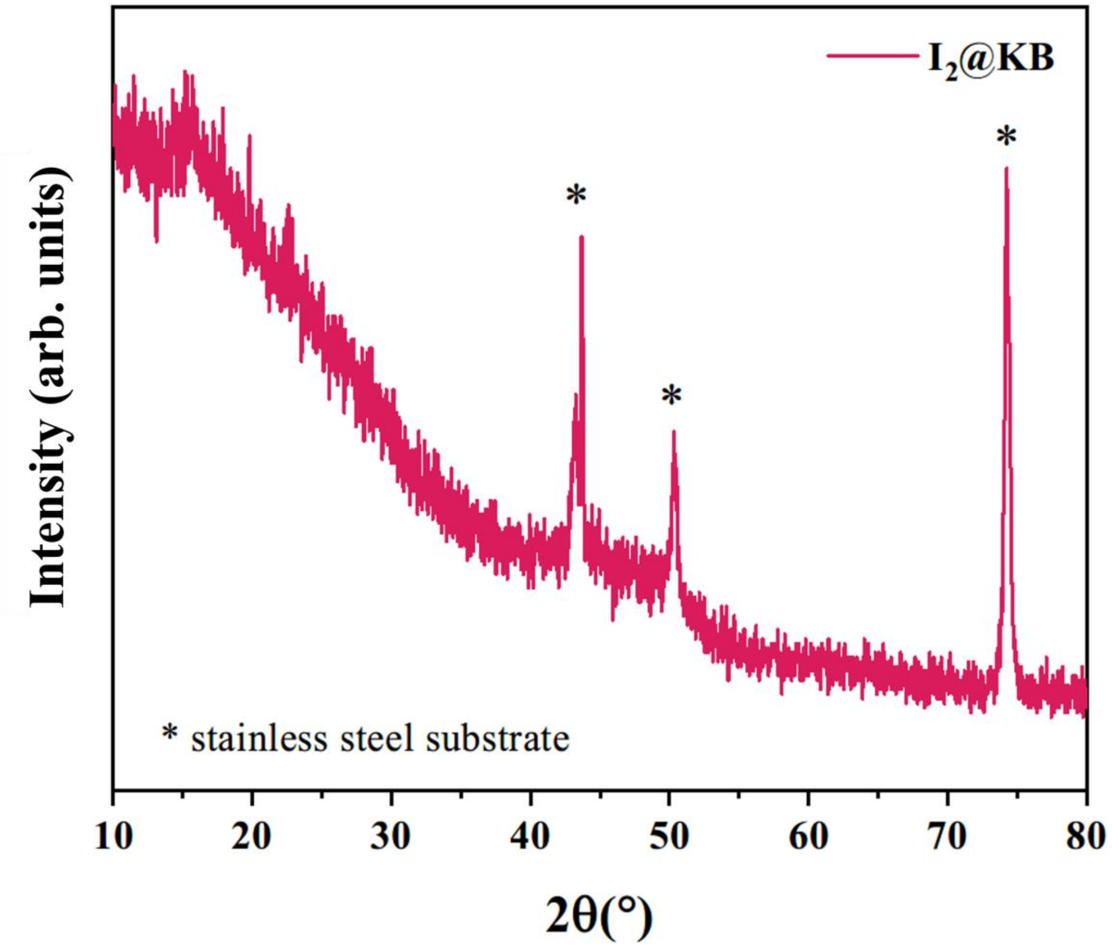

Supplementary Figure 11 | Structural characterization of carbon-host iodine composite. XRD pattern of ball milled I_2@KB powder, showing an amorphism that the crystallinity of I_2 was destroyed by the ball milling process. The peaks marked with asterisk were from stainless steel substrate of the XRD holder.

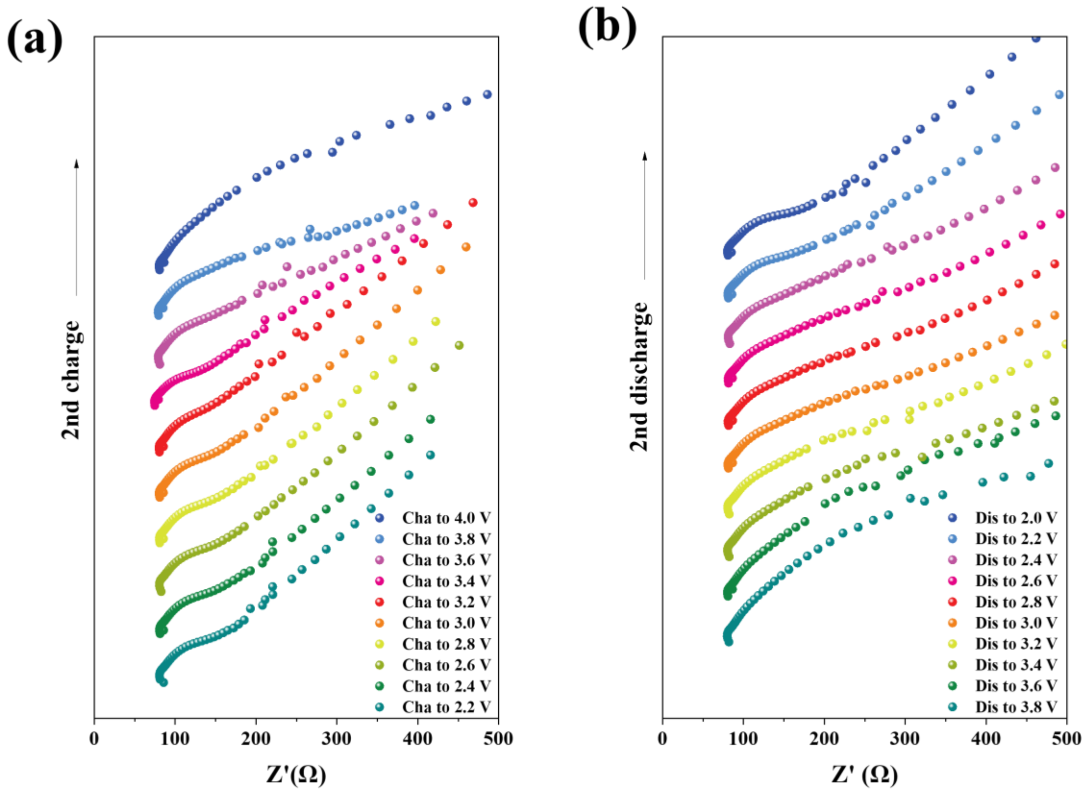

Supplementary Figure 12 | In-situ impedance evolution during electrochemical cycling. (a) EIS evolution of I_2/LIC7.2 electrode-based ASS Li||I_2 battery during discharge at the second cycle. (b) EIS evolution of I_2/LIC7.2 electrode-based ASS Li||I_2 battery during charge at the second cycle. The potentials are versus Li^+/Li.

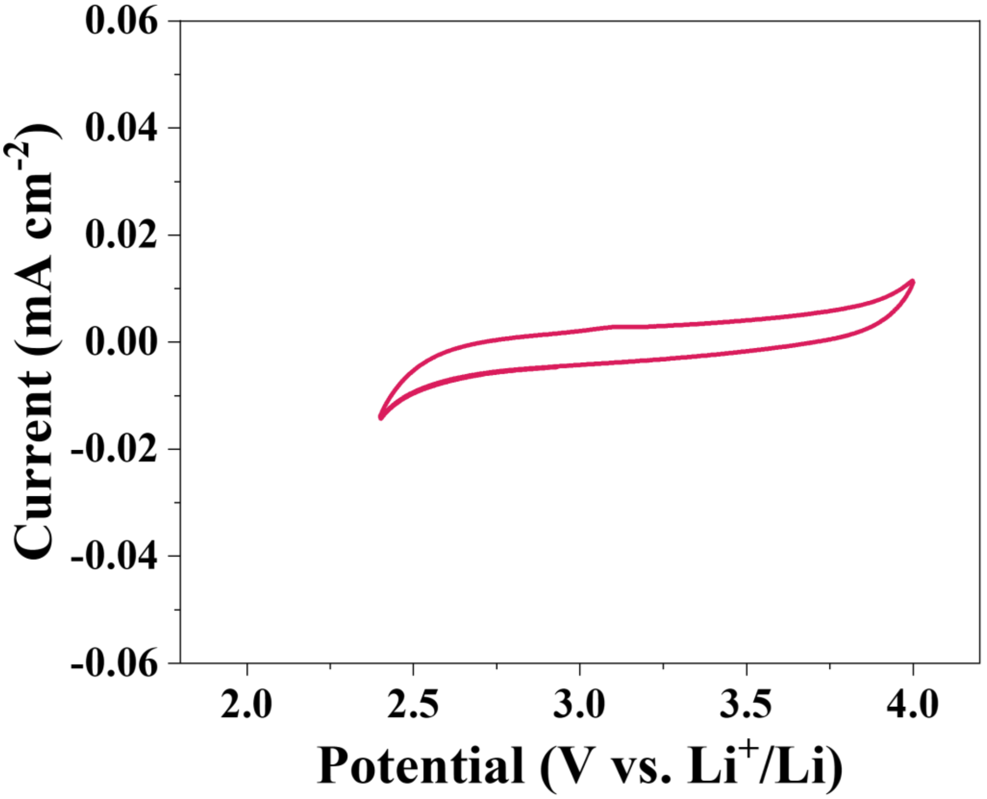

Supplementary Figure 13 | Electrochemical redox behavior of LIC7.2 electrolyte. CV curve of the LIC7.2 electrode with a sweeping voltage range of 2.4 ~ 4 V versus Li^+/Li at a sweeping rate of 0.1 mV s^{-1}.

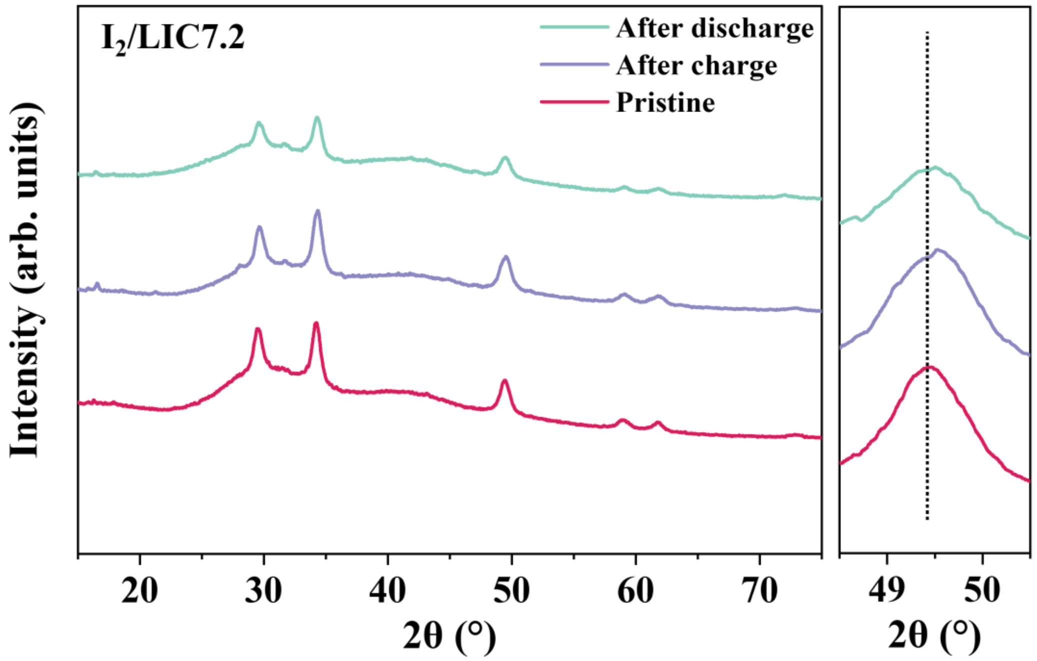

Supplementary Figure 14 | Structural evolution of LIC7.2 catholyte analysis during battery operation. XRD patterns of the I_2/LIC7.2 electrodes at pristine, charged and discharged states. A slight shift is observed for the charged and discharged sample, indicating a delithiation/lithiation behavior of the LIC7.2 during discharge/charge.

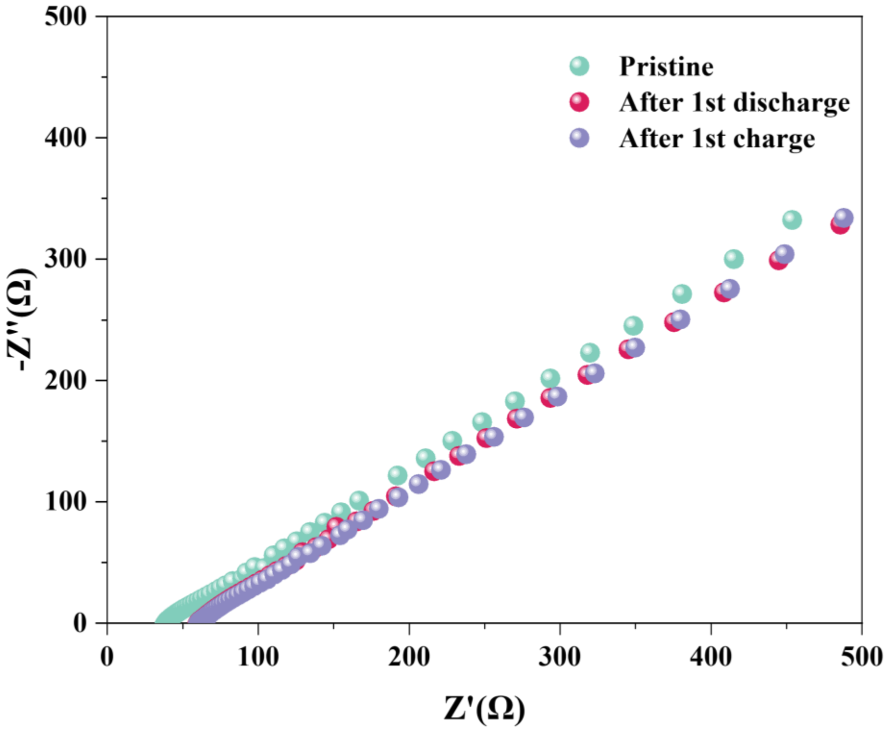

Supplementary Figure 15 | Interfacial resistance monitoring during initial cycle. Nyquist plots of the LIC7.2 electrode-based battery at pristine state, after 1st discharge and after 1st charge. The overall battery resistance kept similar during the first cycle, indicating that the redox of LIC7.2 did not destroy its efficient Li-ion transport capability.

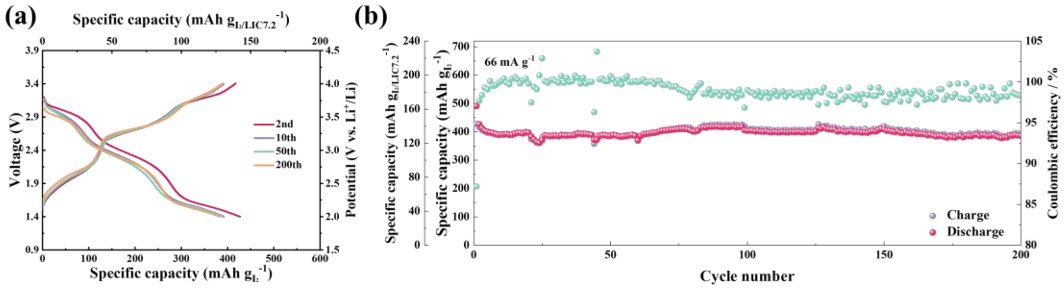

Supplementary Figure 16 | Long-term electrochemical performance evaluation under low mass loading. (a) Charge/discharge curves of the battery at 2nd, 10th, 50th and 200th cycle with a mass loading of 0.5 mg cm^{-2}. (b) Cycling stability with the corresponding coulombic efficiency of the battery at 66 mA g^{-1} and room temperature (25°C). The I_2 mass loading is 0.5 mg cm^{-2}.

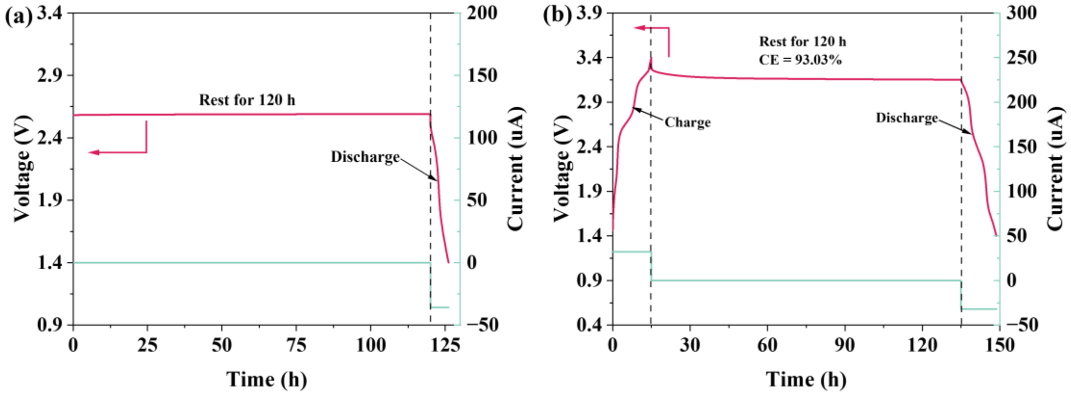

Supplementary Figure 17 | Storage stability assessment under different conditions. Self-discharge performance of the ASS Li||I_2 battery at (a) OCV and (b) after charging. Test was operated at 44 mA g^{-1} at 25 °C. The I_2 mass loading is 1 mg cm^{-2}.

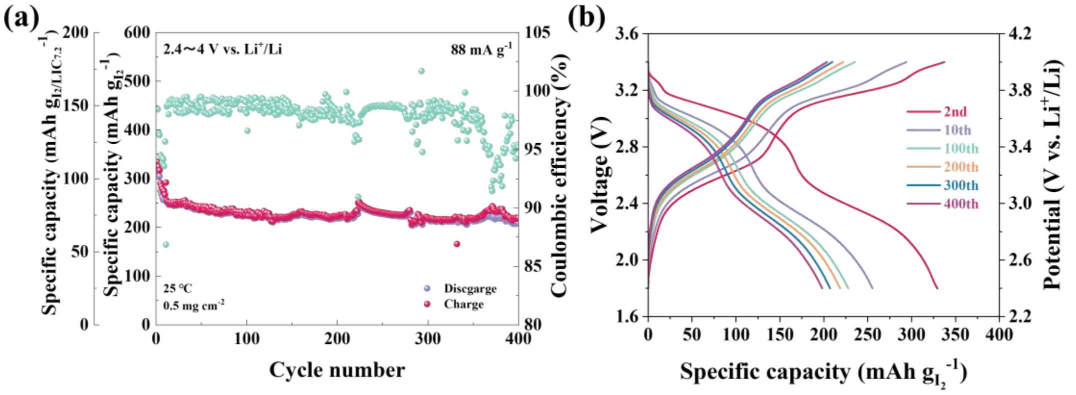

Supplementary Figure 18 | Extended cycling performance within optimized voltage window. (a) Cycling stability and (b) charge/discharge curves of I_2/LIC7.2 battery cycling at the voltage range of 2.4~4 V versus Li^+/Li at room temperature (25°C) with a mass loading of 0.5 mg cm^{-2}. The battery was charged/discharged at 44 mA g^{-1} for first 5 cycles for activation and then at 88 mA g^{-1} for long-term cycling test. The redox capacity of LIC7.2 was excluded during the cycling, as well as partial of the I^-/I_2 conversion.

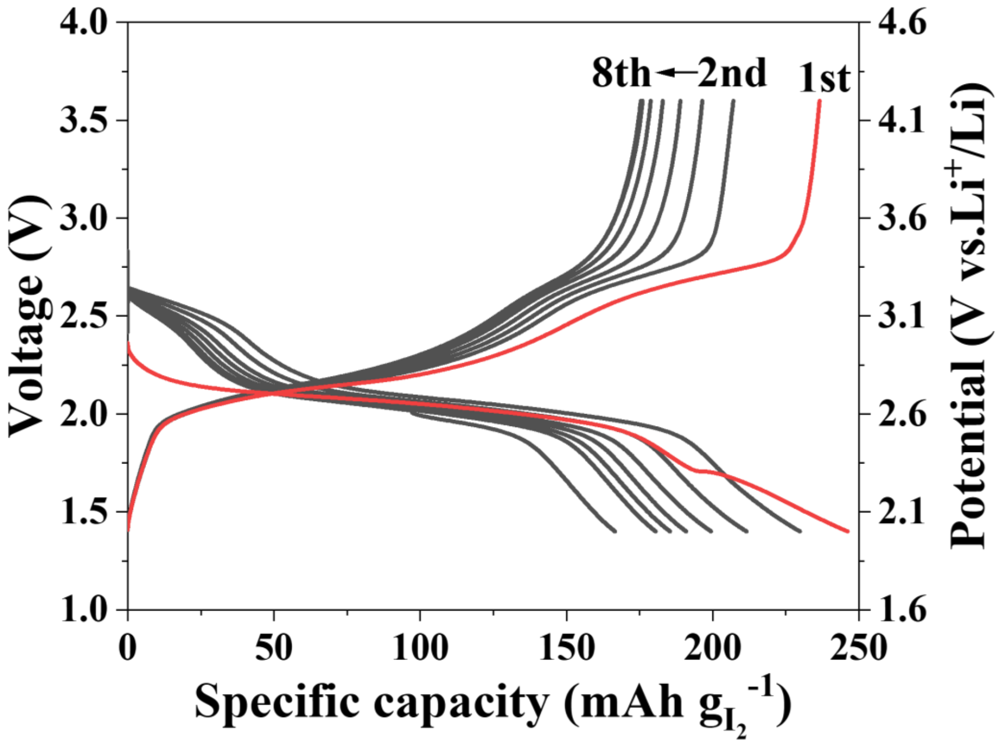

Supplementary Figure 19 | Comparative electrochemical behavior of alternative electrolyte system. Charge/discharge curves of I_2/LYB battery cycling under 22 mA g^{-1} at room temperature (25°C). The specific capacity is calculated based on the I_2 mass. The I_2 mass loading is 0.5 mg cm^{-2}.

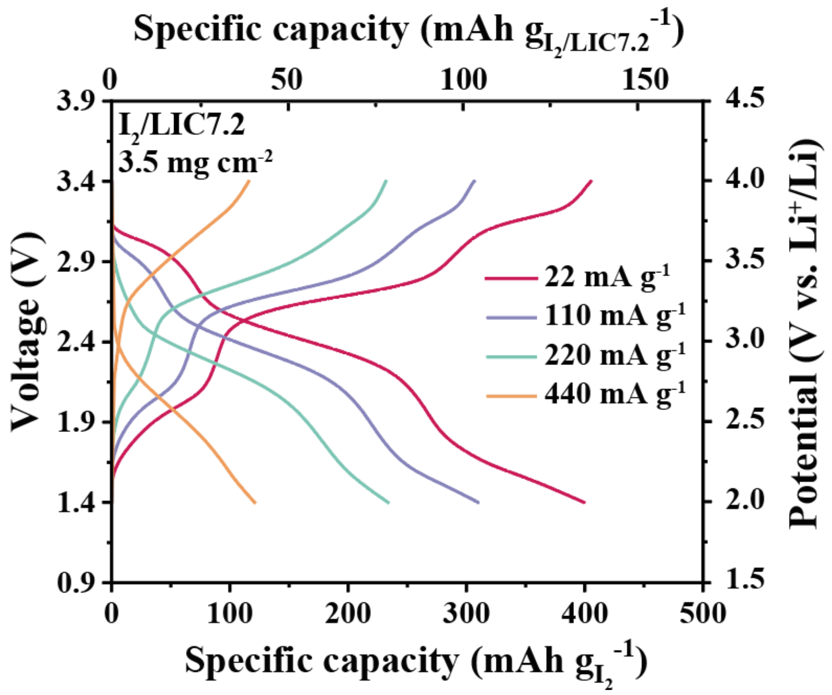

Supplementary Figure 20 | Rate capability analysis at high mass loading. (a) Charge/discharge curves of I_2/LIC7.2 battery cycling under different rates at room temperature (25℃). The mass loading was 3.5 mg cm^{-2}, corresponding to an areal capacity of 1.42 mAh cm^{-2}.

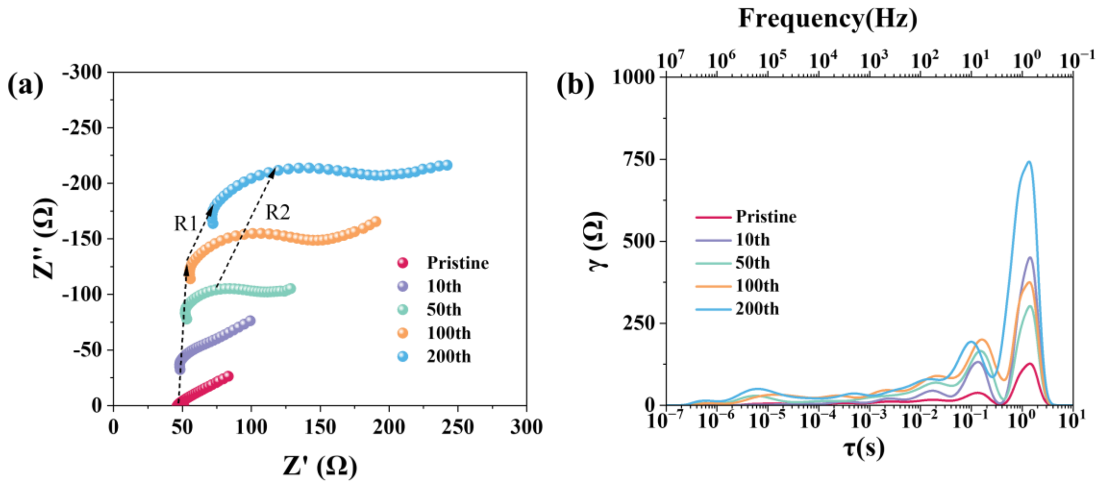

Supplementary Figure 21 | Long-term interface impedance evolution. (a) The EIS measurements of the I_2/LIC7.2 battery with a mass loading of 3.5 mg cm-2 at pristine state and after 10, 50, 100 and 200 cycles. R1 represents the electrolyte resistance while R2 represents the electrode/solid electrolyte interfacial resistance. (b) The DRT spectra calculated from the EIS measurements in (a).

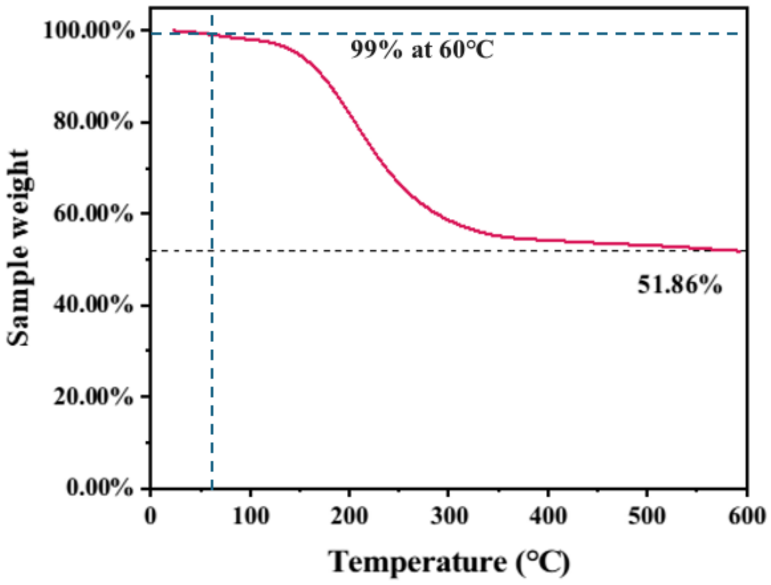

Supplementary Figure 22 | Thermal stability analysis of carbon-host iodine. Thermogravimetric analysis curves of I_2@KB. The weight ratio of I_2 was 48.14% in I_2@KB sample. While bare I_2 easily sublimates at temperature higher than 40°C, the effective trapping of I_2 inside the pores of KB avoids I_2 loss at 60°C in the I_2@KB. The I_2@KB still keeps 99% of its original mass at 60°C.

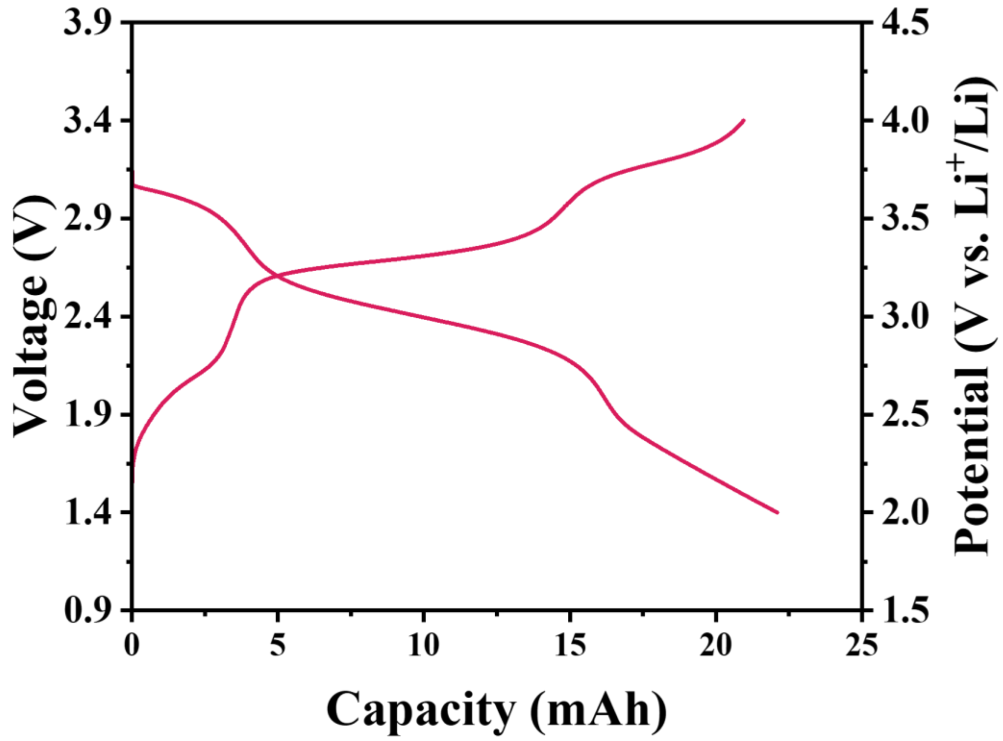

Supplementary Figure 23 | Scalability demonstration through pouch cell performance. The discharge/charge voltage profile of the ASS Li||I_2 pouch cell (40 × 40 mm film battery in a 60 × 60 case) with a capacity of 22 mAh. The pouch cell was operated within the voltage range of 2~4 V versus Li^+/Li at 22 mA g^{-1} and 25 °C.

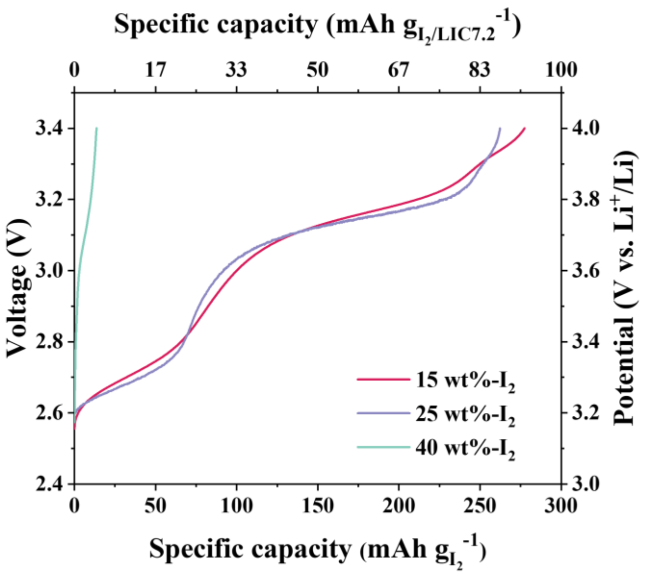

Supplementary Figure 24 | Mass loading optimization study. The voltage profile of ASS Li||I_2 battery with varying I_2 content in the composite positive electrode. At relatively low active material content of 15 wt% and 25 wt%, the I_2/LIC7.2 electrode shows full utilization of the I_2/I^+ redox capacity. When the active material content increases to 40 wt%, the battery only exhibits a very limited activation of the I_2/I^+ redox. This is attributed to the insufficient I-Cl coordination environment near the I_2 particles, as well as a poor ionic conductivity. All tests were performed with a controlled I_2 mass loading of 0.5 mg cm-2. All tests were performed over a range from open circuit voltage to 4 V versus Li^+/Li at 25 °C.

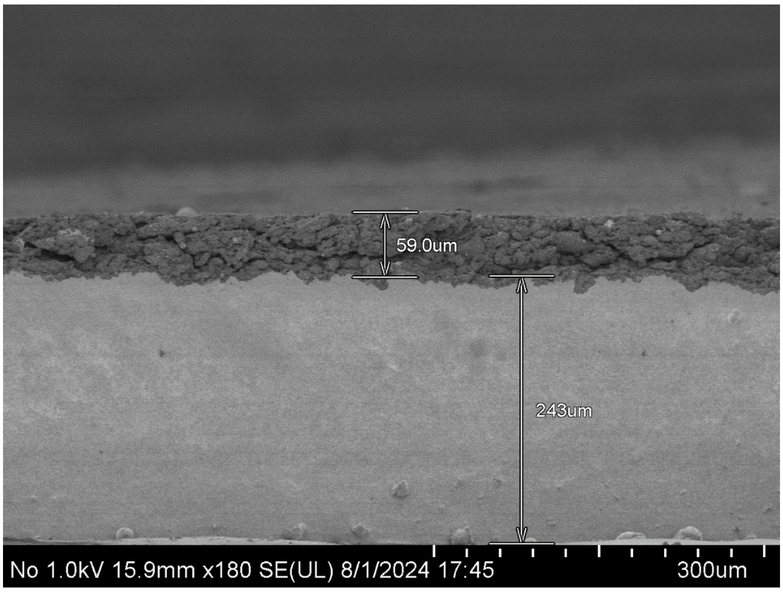

Supplementary Figure 25 | Interface engineering analysis through cross-sectional imaging. Cross-section scanning electron microscopy image of the laminated I_2/LIC7.2 electrode and LIC6 solid electrolyte layer.

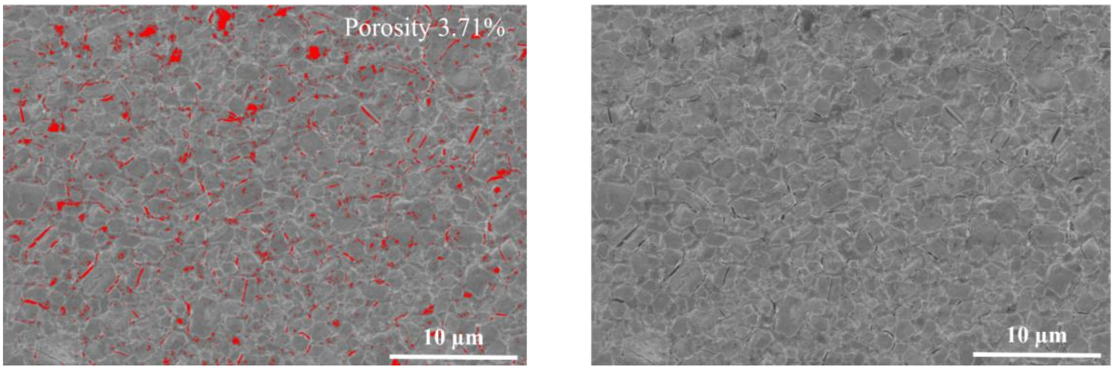

Supplementary Figure 26 | Microstructural characterization of solid state electrolyte layer. Scanning electron microscopy image of the pressed LIC6 solid electrolyte layer. The porosity is calculated to be 3.71%.

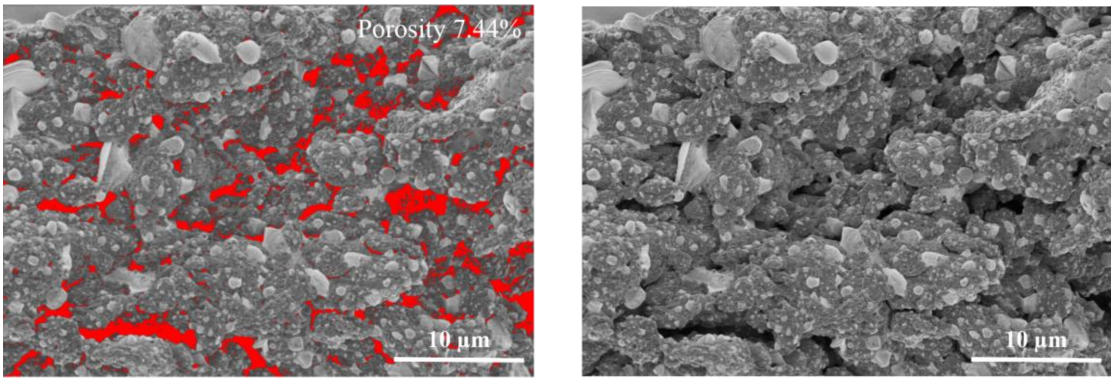

Supplementary Figure 27 | Electrode morphology and porosity analysis. Scanning electron microscopy image of the pressed I_2/LIC7.2 electrode. The porosity is calculated to be 7.44%.

Table S1 | Rietveld analysis results for the XRD pattern of Li_3InCl_6.

**a =** 6.4204(7) **Å** **b =** 11.0746(5) **Å** **c =** 6.3936(1) **Å** **V =** 428.22(6) **Å**^{3} **Space group** C2/m						
**Site**	**x**	**Y**	**Z**	**Fractional Occupancy**	**Uiso**	**Wyckoff site**
Cl1	0.27175	0.16829	0.25479	1	0.01	8j
Cl2	0.77727	0.00000	0.27863	1	0.01	4i
In1	0.00000	0.16732	0.50000	0.53	0.01	4h
Li1	0.00000	0.27492	0.00000	1	0.01	4g
Li2	0.00000	0.00000	0.00000	1	0.01	2a

Table S2 | Rietveld analysis results for the XRD pattern of $Li_{4.2}InCl_{7.2}$.

a = 6.4022(0) **Å** **b =** 11.0627(8) **Å** **c =** 6.3747(6) **Å**
V = 424.56(7) **Å**^{3} **Space group** C2/m

Site	x	Y	Z	Fractional Occupancy	Uiso	Wyckoff site
Cl1	0.24216	0.16333	0.25462	0.875	0.01	8j
Cl2	0.72525	0.00000	0.26660	0.875	0.01	4i
In1	0.00000	0.17641	0.50000	0.375	0.01	4h
Li1	0.00000	0.27841	0.00000	1	0.01	4g
Li2	0.00000	0.00000	0.00000	1	0.01	2a

Table S3 | Element ratio of LIC7.2 obtained by SEM.

Element	unn. [wt.%]	C norm [wt.%]	C Atom [at.%]	C Error [wt.%]
Cl	49.54	68.04	87.33	1.83
In	23.28	31.96	12.67	1.04
