# Supplementary material for: Realizing four-electron conversion chemistry for all-solid-state Li||I2 batteries at room temperature
Source: Nat Commun. 2025 Feb 18;16:1723. doi: 10.1038/s41467-025-56932-5 (PMC11836363; doi:10.1038/s41467-025-56932-5)
Supplement: Supplementary file 3 — Description of Additional Supplementary Files [file 41467_2025_56932_MOESM3_ESM.pdf]

Description of Additional Supplementary Files

Supplementary Data 1: structure of LIC matrix used in DFT calculations.

Supplementary Data 2: structure of LIC matrix used in DFT calculations.

Supplementary Data 3: structure of IBr-LYB matrix used in DFT calculations.

Supplementary Data 4: structure of ICl-LIC matrix used in DFT calculations**.**

Supplementary Data 5: structure of I-LIC matrix used in DFT calculations.

Supplementary Data 6: structure of I-LYB matrix used in DFT calculations.
